# A dual role for integrin-linked kinase and β1-integrin in modulating cardiac aging

**DOI:** 10.1111/acel.12193

**Published:** 2014-01-09

**Authors:** Mayuko Nishimura, Caroline Kumsta, Gaurav Kaushik, Soda B Diop, Yun Ding, Jumana Bisharat-Kernizan, Hannah Catan, Anthony Cammarato, Robert S Ross, Adam J Engler, Rolf Bodmer, Malene Hansen, Karen Ocorr

**Affiliations:** 1 Development, Aging and Regeneration Program, Sanford-Burnham Medical Research Institute10901 North Torrey Pines Road, La Jolla, CA, 92037, USA; 2 Sanford Consortium for Regenerative Medicine, University of California at San Diego2880 Torrey Pines Scenic Drive, La Jolla, CA, 92037, USA; 3 School of Medicine, VA San Diego Healthcare System, University of California at San Diego3350 La Jolla Village Drive, Cardiology Section 111A, San Diego, CA, 92161, USA; 4 Division of Cardiology, Department of Medicine, School of Medicine, Johns Hopkins UniversityBaltimore, MD, 21287, USA

**Keywords:** *Drosophila*, *Caenorhabditis elegans*, arrhythmia, cardiomyopathy, cell adhesion, heart failure, senescence, *ilk*, *myospheroid*, *parvin*, *paxillin*, *pinch*, *talin*

## Abstract

Cardiac performance decreases with age, which is a major risk factor for cardiovascular disease and mortality in the aging human population, but the molecular mechanisms underlying cardiac aging are still poorly understood. Investigating the role of integrin-linked kinase (*ilk*) and β1-integrin (*myospheroid, mys*) in *Drosophila*, which colocalize near cardiomyocyte contacts and Z-bands, we find that reduced *ilk* or *mys* function prevents the typical changes of cardiac aging seen in wildtype, such as arrhythmias. In particular, the characteristic increase in cardiac arrhythmias with age is prevented in *ilk* and *mys* heterozygous flies with nearly identical genetic background, and they live longer, in line with previous findings in *Caenorhabditis elegans* for *ilk* and in *Drosophila* for *mys*. Consistent with these findings, we observed elevated β1-integrin protein levels in old compared with young wild-type flies, and cardiac-specific overexpression of *mys* in young flies causes aging-like heart dysfunction. Moreover, moderate cardiac-specific knockdown of integrin-linked kinase (ILK)/integrin pathway-associated genes also prevented the decline in cardiac performance with age. In contrast, strong cardiac knockdown of *ilk* or ILK-associated genes can severely compromise cardiac integrity, including cardiomyocyte adhesion and overall heart function. These data suggest that *ilk/mys* function is necessary for establishing and maintaining normal heart structure and function, and appropriate fine-tuning of this pathway can retard the age-dependent decline in cardiac performance and extend lifespan. Thus, ILK/integrin-associated signaling emerges as an important and conserved genetic mechanism in longevity, and as a new means to improve age-dependent cardiac performance, in addition to its vital role in maintaining cardiac integrity.

## Introduction

With age, heart function declines and the prevalence of heart disease is dramatically increased. For example, the incidence of heart failure and atrial fibrillation is markedly increased in the elderly (Lakatta & Levy, 2003; Roger *et al*., 2011), which suggests that aging *per se* is a major risk factor for heart disease. It is well known that the heart undergoes many age-related functional and structural changes (Khan *et al*., 2002; Bernhard & Laufer, 2008). However, the control mechanisms of cardiac-intrinsic aging, and their coordination with organismal aging, remain elusive.

Cardiac decline with age and many specific age-related changes occurring in the human heart have also been observed in a variety of other species. Therefore, insights from model organisms, such as *Drosophila*, the simplest (genetic) model system with a heart (Bier & Bodmer, 2004), will likely provide valuable clues for understanding the cellular and molecular mechanisms involved in cardiac aging (Dai *et al*., 2010; Nishimura *et al*., 2011). *Drosophila* has a short lifespan that makes it an ideal model system for studying the genetic underpinnings of aging. Although the linear heart tube of *Drosophila* is much less complex than the mammalian heart, its development and functional characteristics are remarkably conserved (Bodmer, 1995; Olson, 2006; Ocorr *et al*., 2007; Cammarato *et al*., 2008; Bodmer & Frasch, 2010). With age, the fly heart also shows features similar to mammals, including humans, with respect to structural alterations and propensity toward arrhythmias (Wessells *et al*., 2004; Ocorr *et al*., 2007; Cammarato *et al*., 2008; Taghli-Lamallem *et al*., 2008; Fink *et al*., 2009). At young ages, surgically exposed fly hearts show a regular myogenic beating pattern. As flies age, these heartbeats become less regular and show increased arrhythmias, which are reminiscent of the increased incidence of atrial fibrillation in elderly humans. Thus, many fundamental aspects of cardiac aging seem to be conserved (Bodmer & Frasch, 2010; Dai *et al*., 2010), as is organismal aging (Kenyon, 2010).

Integrins are major adhesive transmembrane receptors that bind to the extracellular matrix (ECM). Their activation affects cytoskeletal remodeling and other intracellular signaling pathways (Delon & Brown, 2007). Integrin signaling and the link between integrins and the cytoskeleton are mediated by many proteins, including integrin-linked kinase (ILK), Talin, and focal adhesion kinase (FAK; Geiger *et al*., 2001). Mammalian ILK is a putative serine/threonine kinase, originally identified as a binding partner for the cytoplasmic tail of β1-integrin (Hannigan *et al*., 1996,2007). ILK also binds to the adapter proteins Parvin, Pinch, and Paxillin (Pax), thereby providing an integrin signaling platform (Legate *et al*., 2006). *Drosophila* has single orthologues of ILK, Parvin, Pinch, and Pax (Legate *et al*., 2006). In *Drosophila*, *ilk* homozygous mutants are embryonic lethal and show severe muscle attachment defects (Zervas *et al*., 2001). ILK is also essential for development in mouse and *Caenorhabditis elegans* (MacKinnon *et al*., 2002; Sakai *et al*., 2003). Remarkably, RNAi-induced reduction in ILK, or the ILK binding partner Parvin, causes increased longevity in *C. elegans* (Hansen *et al*., 2005; Curran & Ruvkun, 2007). These findings suggest that in contrast to complete loss of *ilk*, which is deleterious for organismal development, moderate *ilk* knock-down (KD) extends lifespan in *C. elegans*. Interestingly, heterozygous *Drosophila* mutants for β1-integrin (*mys*) also have an increased lifespan (Goddeeris *et al*., 2003). Therefore, reduced β1-integrin/ILK signaling may also be beneficial for cardiac-specific aging. Interestingly, overexpression of *ilk* in rat cardiac fibroblasts induces cellular senescence, whereas inhibition of *ilk* prevents senescence-related changes in these cells (Chen *et al*., 2006). In contrast, conditionally targeted knockout of *ilk* in the mouse heart causes left ventricle dilation, heart failure, disaggregation of cardiac tissue, leading to sudden death (White *et al*., 2006). Taken together, these results indicate a critical role for *ilk* in establishing and maintaining heart contractility. Thus, we hypothesize that *ilk* has a dual role in the heart, one that modulates cardiac aging and one that maintains the heart’s structural integrity.

In this study, we demonstrate that reduced integrin/ILK ameliorates the effects of normal cardiac and organismal aging in *Drosophila*. *ilk* and *mys* heterozygotes not only live longer, but their hearts perform better at old age than wild-type controls, similar to young flies. Moreover, moderate cardiac-specific KD of integrin/ILK*-*associated genes, *pax, parvin*, *talin,* and *pinch,* also prevents the decline of heart performance with age. Conversely, cardiac overexpression of *mys* causes a senescent-like phenotype in young flies. These findings suggest that the accumulation of β1-integrin at an older age may mediate in part the declining heart function and that a moderate reduction in integrin/ILK activity maintains youthful heart function with age. In contrast, more severe cardiac-specific KD of *ilk* and other ILK-associated components leads to a higher incidence of cardiac arrhythmia already in young flies, which is accompanied by defective cellular adherence of the cardiomyocytes. Thus, severely compromised integrin/ILK pathway function is detrimental for the heart, but fine-tuned moderate reduction maintains youthful cardiac performance, suggesting a dual role for this complex in regulating cardiac integrity and aging.

## Results

### *ilk* heterozygous mutants have extended lifespan in *Drosophila*

**Figure 1:**
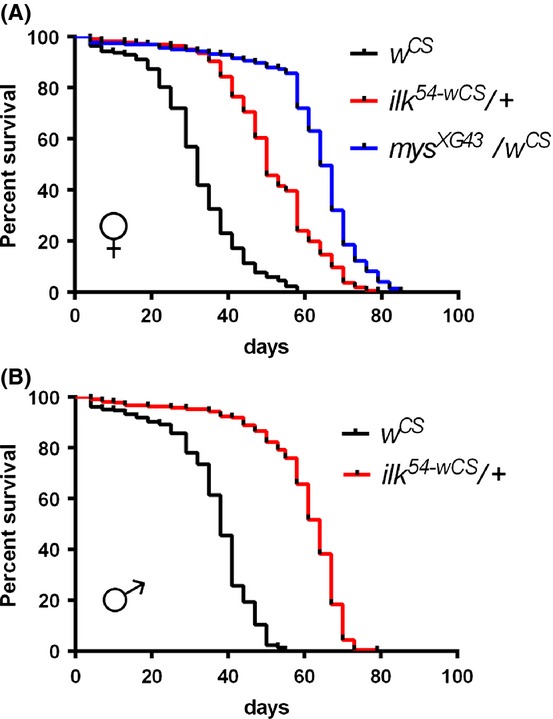
Integrin-linked kinase (*ilk*) heterozygous mutants have increased lifespan. The lifespan of *ilk*^*54-*^^*wCS*^/+ (red), *mys*^*XG43*^*/w*^*CS*^ (blue), and *w*^*CS*^ (control, black) flies was examined. *ilk*^*54*^ and *mys*^*XG43*^ were introgressed into the *w*^*CS*^ wild-type background for six generations. Top, the survival curves of female *w*^*CS*^ (mean 32 days, *N* = 222)*, ilk*^*54-*^^*wCS*^/+ (mean 51 days, *N* = 217), and *mys*^*XG43*^/+ (mean 62 days, *N* = 222) flies. Bottom, the survival curves of male *w*^*CS*^ (mean 36 days, *N* = 222) and *ilk*^*54-*^^*wCS*^/+ (mean 59 days, *N* = 207) flies. As *mys* is X-linked and hemizygous mys/Y flies are lethal, males could not be examined. Both male and female *ilk*^*54-*^^*wCS*^/+ flies, and female *mys*^*sXG43*^/+ flies lived longer than the controls (*w*^*CS*^). For statistics, see Table 1. Survival curves of trial 2 in Table 1 are shown.

As the RNAi-mediated KD of *ilk* extends lifespan in *C. elegans* (Hansen *et al*., 2005; Curran & Ruvkun, 2007; Kumsta *et al*., 2014), we wondered whether reduced *ilk* expression is also beneficial to longevity in *Drosophila*. As lifespan can be significantly modulated by genetic background (Grandison *et al*., 2009), we first backcrossed *ilk*^*54*^ mutants (premature stop codon; Zervas *et al*., 2011; see Experimental procedures) to the wild-type control strain, *w*^*CS*^ (Cook-Wiens & Grotewiel, 2002) for six generations. The resulting backcrossed lines are referred to as *ilk*^*54-wCS*^. We found that both female and male *ilk*^*54-wCS*^ heterozygous mutants (*w*^*CS*^;*ilk*^*54-wCS*^*/+*) show extended lifespan compared with their *w*^*CS*^ controls (Table 1, Fig. 1).

**Table 1:** Lifespan extension in *ilk*^*54-wCS*^/+ and *mys*^*XG43*^/*w*^*CS*^ flies

	Sexes	Genotypes	*N*	Median survival	% extension	*P*
Trial1	Female	*w*^*CS*^	153	45		
		*ilk*^*54-wCS*^/+	110	66	37	< 0.0001
		*mys*^*XG43*^/*w*^*CS*^	128	66	44	< 0.0001
	Male	*w*^*CS*^	150	45		
		*ilk*^*54-wCS*^/+	107	66	56	< 0.0001
Trial2	Female	*w*^*CS*^	222	32		
		*ilk*^*54-wCS*^/+	217	54	60	< 0.0001
		*mys*^*XG43*^/*w*^*CS*^	222	64	95	< 0.0001
	Male	*w*^*CS*^	222	38		
		*ilk*^*54-wCS*^/+	207	64	63	< 0.0001

Lifespan was examined in *ilk*^*54-wCS*^/+ and *mys*^*XG43*^/*w*^*CS*^ flies first on the smaller scale (Trial 1) and then on larger scale (Trial 2). *mys* gene is on X chromosome; thus, only female heterozygotes with null *mys* mutation (*mys*^*XG43*^) can be tested. % extension of mean lifespan compared with *w*^*CS*^ is shown. *P* values were obtained from log-rank analysis (Mantel–Cox test).

β1-integrin interacts with ILK (Hannigan*et al*.,^1996^,^2007^), and homozygous mutants (*mys*) have embryonic muscle phenotypes similar to *ilk* mutants (Zervas *et al*., 2001). In addition, *mys* heterozygotes have previously been reported to exhibit an extended mean lifespan (Goddeeris *et al*., 2003). Thus, we re-examined the lifespan of *mys*^*XG43*^ heterozygotes (also the *w*^*CS*^ genetic background contains small deletion and premature stop codon; Goddeeris *et al*., 2003; see Experimental procedures) and confirmed that *mys*^*XG43*^/*w*^*CS*^ flies have a significantly extended lifespan, similar to *ilk*^*54-wCS*^*/+* flies (Table 1, Fig. 1), in terms of both maximum and mean lifespan (Fig. 1). Together, these data suggest a conserved role of the integrin/ILK pathway in organismal aging.

### ILK is localized to cell–cell contact sites and Z-disks in adult hearts

**Figure 2:**
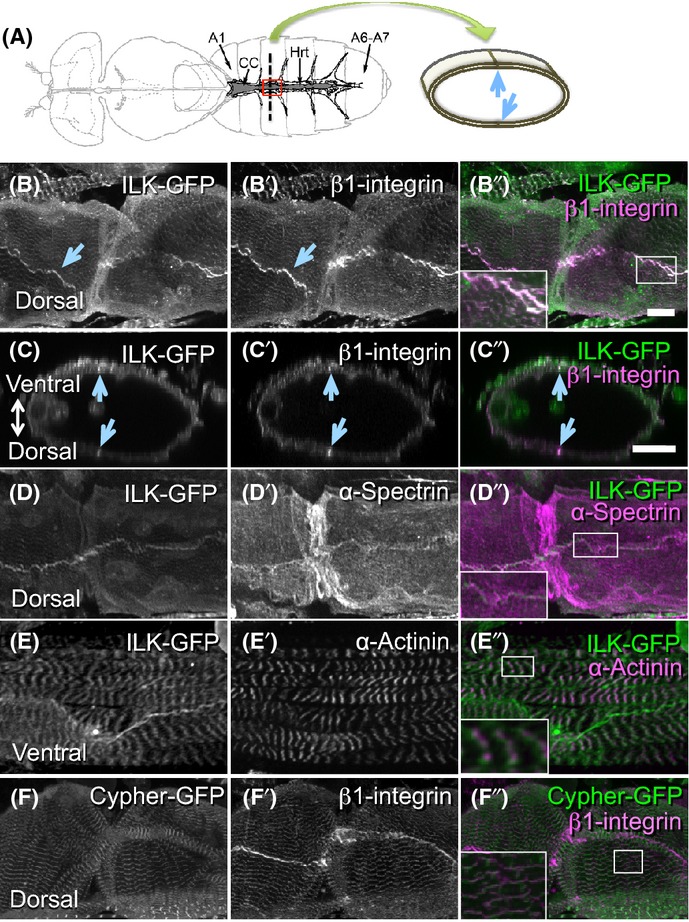
Integrin-linked kinase (ILK) and β1-integrin are localized to cell–cell contact sites and Z-disks in fly hearts. (A) Left, a schematic diagram of the adult fly heart (adopted from Cammarato *et al*., 2008). Hrt, fly heart tube; CC, conical chamber; A1, abdominal segment 1; A6-A7, abdominal segments 6 and 7. The region outlined in red corresponds to the area shown in B, D, E, and F. Right, a schematic diagram of a cross section of the heart tube along the dotted line in the left panel. The circumference of the heart tube is composed of two myocardial cells. Myocardial cell–cell contact sites are pointed out by arrows. (B-B”) Dorsal views of the heart tube. Both ILK (B and green in B”) and β1-integrin (B’ and magenta in B”) are expressed in adult hearts and colocalize. (C-C”) Optical cross sections from B-B” show circumference of the heart tube. The ventral side is to up. Prominent signals of ILK and β1-integrin are indicated by arrows in B–C”. (D-D”) ILK (D and green in D”) was found at the cell membrane labeled with α-spectrin (D’ and magenta in D”). (E-E”) Ventral views of the heart tube. ILK (E and green in E”) was also localized to Z-disks marked by α-actinin (E’ and magenta in E”). (F-F”) Dorsal views of the heart tube. β1-integrin (F and magenta in F”) is also associated with Z-disks marked by cypher-GFP (F’ and green in F”). All the images in B–F” are in abdominal segment 3 region. Insets in B”, D”, E”, and F” are higher magnification images corresponding to the each rectangle in the respective figures. Bars: 20 μm.

To examine *ilk* expression in the *Drosophila* heart (Fig. 2A), we used a genomic *ilk*-*GFP* fusion rescue construct that contains the putative *ilk* promoter, enhancers, and *ilk* transcription unit. This transgene fully rescues the embryonic *ilk* mutant defects previously reported (Zervas *et al*., 2001). Similar to the abundant expression of *ilk* in human hearts (Hannigan*et al*.,^1996^,^2007^), *ilk-GFP* was also expressed in the adult *Drosophila* heart and colocalized with β1-integrin (Fig. 2A–C). Interestingly, ILK-GFP and β1-Integrin prominently accumulated at or near cell–cell junctions at both dorsal and ventral sides of cardiomyocytes (Fig. 2B,C, arrows), where it coincides with the cytoskeletal protein α-spectrin (Fig. 2D; Pesacreta *et al*., 1989). This suggests that ILK and β1-integrin concentrate at the plasma membrane that contacts adjacent cardiomyocytes within the heart tube (see Fig. 2A). ILK-GFP accumulation was also found to colocalize with α-actinin, a sarcomeric Z-disk marker (Fig. 2E). β1-integrin also colocalizes with cypher-GFP, another Z-disk marker in myocardial cells (Fig. 2F).

### *ilk and mys* heterozygotes do not exhibit age-related increases in arrhythmias

**Figure 3:**
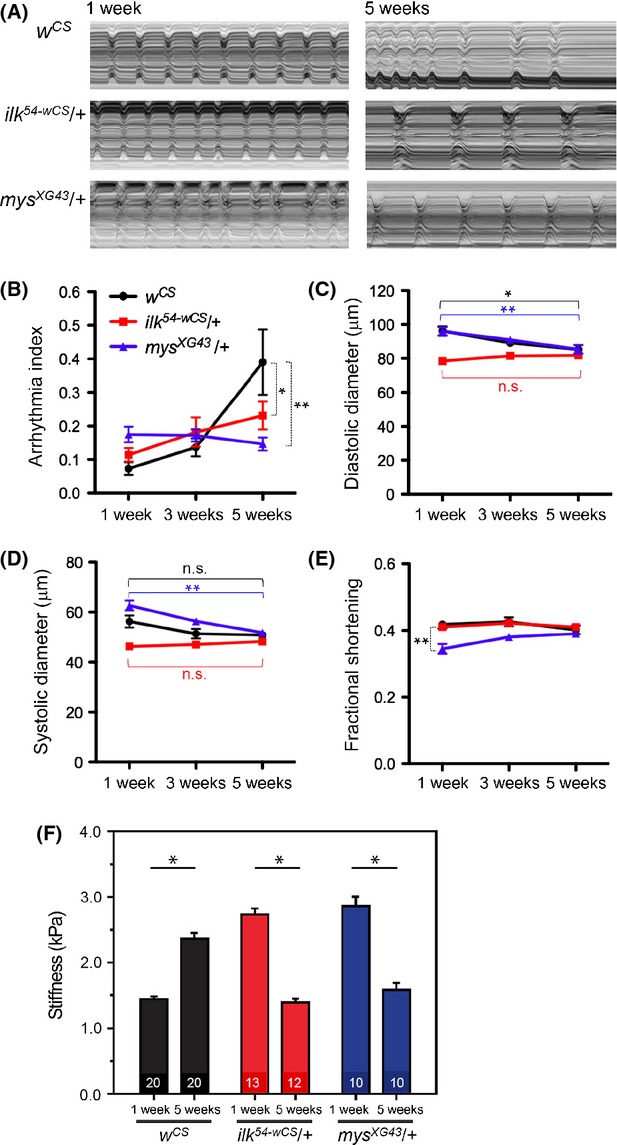
Changes in heart function with age are attenuated in *ilk* and *mys* heterozygous mutants. (A) M-mode traces from 10 s movies show heartwall movements in 1-week-old (left) and 5-week-old (right) hearts. Top, *w*^*CS*^. Middle, *ilk*^*54-*^^*wCS*^/+. Bottom, *mys*^*XG43*^/+. The incidence of arrhythmia is increased with age in wild-type controls (top panels, see also Ocorr *et al*., 2007), but 5-week-old *ilk*^*54-*^^*wCS*^/+ hearts (middle right) and *mys*^*XG43*^/+ hearts (bottom right) present more regular beating patterns than 5-week-old *w*^*CS*^ hearts (top right). (B–E) Plots of physiological parameters of *w*^*CS*^(●), *ilk*^*54-*^^*wCS*^/+(■), and *mys*^*XG43*^/+ (▲) at 1, 3, and 5 weeks of age. 20–30 flies were used for each data point. (B) Incidence in heartbeat irregularities indicated by the arrhythmia index (AI) represents the standard deviation of heart period normalized to the median value for each fly (Fink *et al*., 2009). Arrhythmia index increases with age in wild-type *w*^*CS*^ hearts (*P* < 0.001 for both 3 and 5 weeks), as previously reported for other wild-type strains (Ocorr *et al*., 2007). Notably, 5-week *ilk*^*54-*^^*wCS*^/+ and *mys*^*XG43*^/+ flies had lower AI compared with the age-matched *w*^*CS*^ flies. *P < 0.01, **P < 0.001, two-way ANOVA, see Experimental procedures. (C, D) Diastolic diameter decreases with age in *w*^*CS*^, and diastolic and systolic diameters decrease *mys*^*XG43*^/+ flies, but not in *ilk*^*54-*^^*wCS*^/+ flies. *P < 0.01, **P < 0.001, two-way ANOVA. (E) Plots of fractional shortening for *w*^*CS*^, *ilk*^*54-*^^*wCS*^/+, and *mys*^*XG43*^/+ flies. 1-week-old *mys*^*XG43*^/+ flies had a significantly lower fractional shortening compared to *w*^*CS*^ and *ilk*^*54-*^^*wCS*^/+. **P < 0.001, two-way ANOVA. (F) Nanoindentation was performed to determine the stiffness (measured in kiloPascal, kPa) of fly heart tubes at the indicated ages and genotypes. Averages and standard errors are shown along with statistical significance, indicated by a Wilcoxon rank-sum test. Number of flies analyzed per condition is indicated at bottom of each bar. *P < 10^−7^. Error bars indicate SEM.

The expression patterns of ILK and β1-integrin in *Drosophila* hearts suggest a possible functional requirement for the β1-integrin/ILK pathway in maintaining normal heart structure and function. To test this possibility, we analyzed cardiac performance of *ilk* and *mys* heterozygotes using high-speed video imaging of semi-intact heart preparations (Ocorr *et al*., 2007). At young ages (1 week old), the control hearts show regular beating patterns (see M-modes in Fig. 3A) and consequently a low arrhythmia index (AI; Fig. 3B). Arrhythmia index is the normalized heart period’s standard deviation similar to the coefficient of variation and is a quantification of the variability in heart period (defined as the period from the beginning of one contraction to the beginning of the next contraction; Fink *et al*., 2009). As wild-type (*w*^*CS*^) flies age, the heart rhythm becomes progressively less regular, as exemplified by increases in AI (Fig. 3A,B; Ocorr *et al*., 2007). In contrast to wild-type, *ilk*^*54-wCS*^/+ flies exhibit a much diminished increase in AI with age (Fig. 3A,B). To gain additional information about heart function, we also measured systolic and diastolic diameters of the hearts from the video images. Wild-type *w*^*CS*^ hearts exhibited a modest decrease in the diastolic as well as systolic diameters with age (Fig. 3C,D), as has previously been observed in other wild-type controls (Cammarato *et al*., 2008). This suggests a tendency of age-related diastolic dysfunction also in flies, as is the case in humans (Lakatta & Levy, 2003), although this parameter is somewhat variable. In contrast, aging *ilk*^*54-wCS*^/+ flies exhibit no significant difference with age in heart tube diameters; however, hearts from these flies are already relatively constricted even at young ages (Fig. 3C,D). Fractional shortening, a measure of cardiac contractility, was preserved in these flies (Fig. 3E).

Similar to *ilk*^*54-wCS*^/+ flies, 5-week-old *mys*^*XG43-wCS*^/+ heterozygotes also exhibited a more regular heart beat pattern, compared with age-matched *w*^*CS*^ controls, manifested as a markedly lower AI, thus abolishing the typical wild-type age-related increase in AI (Fig. 3A,B). However, unlike *ilk*^*54-wCS*^/+ flies, old *mys*^*XG43-wCS*^/+ heterozygotes still showed a modest decrease in diastolic diameter, as *w*^*CS*^ controls (Fig. 3C); thus, diastolic dysfunction was not prevented in this case. Moreover, young *mys*^*XG43-wCS*^ heterozygotes had larger systolic diameters (Fig. 3D) and thus lower fractional shortening (Fig. 3E) compared with controls, suggestive of systolic dysfunction at that age.

We replicated the above findings and confirmed similar trends in different genetic backgrounds: *ilk*^*54*^/+ flies as well as in *mys*^*XG43*^/*+* and *mys*^*1*^/+ fly lines that were not backcrossed to the *w*^*CS*^ background, but were crossed out to *w*^*1118*^, another laboratory wild-type strain (Fig. S1, Supporting Information). Together, the data suggest that reduction in *ilk* or *mys* gene dosage overall attenuates the normally age-dependent changes in heart performance, consistent with an increased lifespan of these flies.

### Reduced *ilk* activity abolishes the age-dependent change in myocardial stiffness

As is observed in human cardiac aging (Lakatta & Levy, 2003), another feature of aging hearts in *Drosophila* is stiffening of the myocardium (measured in kiloPascals, kPa, upon applying external pressure and at the ventral midline of the heart; Kaushik *et al*., 2011, 2012). Because *ilk* heterozygous mutants attenuate or halt the age-dependent changes in heart performance (Fig. 3A,B) and ILK-GFP was found at cell–cell contact sites near the ventral and dorsal midline in the wild-type animal (Fig. 2B–D), we speculated that ILK may be involved in the increase in myocardial stiffness with age. To test this idea, ventral midline stiffness was measured with nanoindentation in *ilk*^*54-wCS*^*/+* and corresponding wild-type controls *w*^*CS*^. Aged *w*^*CS*^ hearts exhibit a stiffening of approximately 65% with age from 1 to 5 weeks of age (Fig. 3F and Fig. S1B). In contrast to *w*^*CS*^, the *ilk*^*54-wCS*^*/+* myocardium did not stiffen with age, but instead started out relatively stiff and then softened with age (Fig. 3F). These results suggest a lack of age-dependent myocardial stiffening in *ilk* heterozygous mutants. Moreover, *mys*^*XG43-wCS*^/+ myocardium also showed lack of stiffening with age, similar to the *ilk*^*54-wCS*^*/+* myocardium (Fig. 3F). Thus, we find that *ilk*^*54-wCS*^*/+* and *mys*^*XG43-wCS*^/+ hearts do not display several measures of cardiac aging, such as increasing AI, diastolic dysfunction (except for *mys*^*XG43-wCS*^/+) and myocardial stiffening.

### β1-integrin protein levels are increased in old flies and overexpression in young fly hearts mimics the effects of cardiac aging

**Figure 4:**
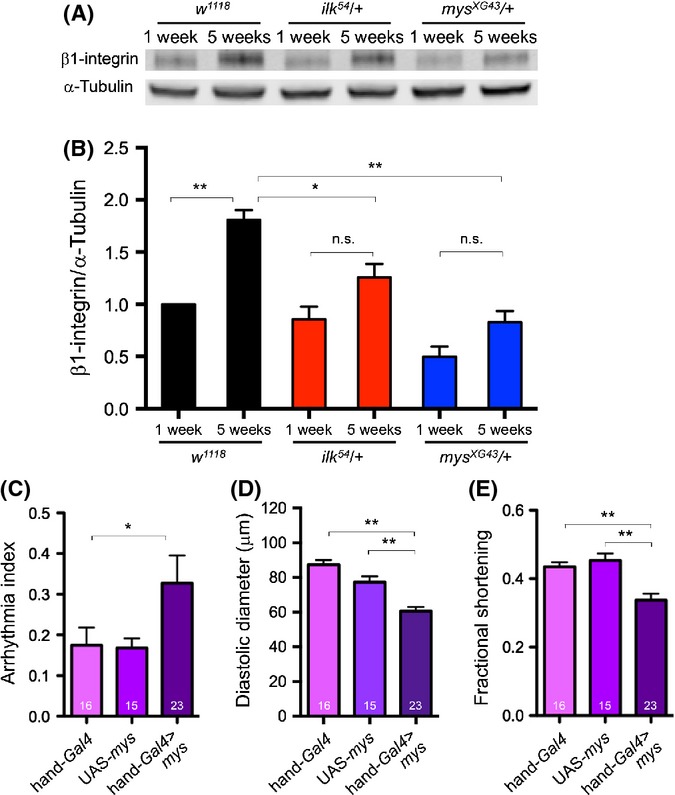
β1-integrin accumulates in old flies and overexpression of *mys* impairs heart function. (A, B) Western blot analysis was carried out with whole fly preps of 1-week and 5-week-old *w*^*1118*^, *ilk*^*54*^/+, and *mys*^*XG43*^/+ flies, using anti-β1-integrin antibody and anti-α tubulin antibody. β1-integrin was almost twofold higher in 5-week-old wild-type control (*w*^*1118*^) compared with 1 week (**P < 0.001, two-way ANOVA). In contrast, β1-integrin accumulation was suppressed in 5-week-old *ilk*^*54*^/+ and *mys*^*XG43*^/+ flies, compared with 5-week-old *w*^*1118*^ (*P < 0.01, **P < 0.001, two-way ANOVA). (B) Triplicate biological samples were analyzed by western blotting and normalizing to tubulin. All data were normalized to the value for *w*^*1118*^ at 1 week. (C, D, and E) Cardiac overexpression of *mys* using hand*-Gal4* driver significantly increased arrhythmia compared with hand-*Gal4* (C), decreased diastolic diameter compared with the controls (hand-*Gal4* and UAS-*mys*) (D), and decreased fractional shortening compared with the controls (hand-*Gal4* and UAS-*mys*) (E) in relatively young flies (3 week old). Sample numbers are indicated at the bottom of each bar. *P < 0.05, **P < 0.001, Kruskal–Wallis test (arrhythmia index) and one-way ANOVA (DD and FS). Error bars are SEM.

As *ilk* and *mys* heterozygous mutants show a much attenuated progression in several age-dependent characteristics (Fig. 3; Fig. S1), we wondered whether *ilk* and *mys* expression levels would be increased in old wild-type flies. Neither *mys* nor *ilk* mRNA levels were increased in 5-week- compared with 1-week-old flies (Fig. S2, Supporting Information). However, when examining β1-integrin protein levels, we found an almost two-fold increase at 5 weeks compared with 1 week (Fig. 4A,B). This increase in β1-integrin protein was attenuated in old *ilk* and *mys* heterozygotes (Fig. 4A,B). To test whether excess *mys* function can induce cardiac aging, we overexpressed *mys* in young hearts and found indeed that it produced an increased AI, reminiscent of old control flies (Fig. 4C). In addition, we observed decreased diastolic diameters and fractional shortening (Fig. 4D,E), suggesting diastolic dysfunction, a characteristic of old flies (Cammarato *et al*., 2008). Although it is possible that overexpression of *mys* may cause dominant-negative effects, the observed phenotype did not resemble a *mys* loss-of-function cardiac phenotype (see also below), but rather the cardiac aging phenotype observed in wild-type flies (Fig. 3B–E). Thus, we suggest that *mys* overexpression in young hearts may possibly accelerate the cardiac aging phenotype.

### Heart-specific *ilk* knockdown causes arrhythmias and impaired adhesion between cardiomyocytes

**Figure 5:**
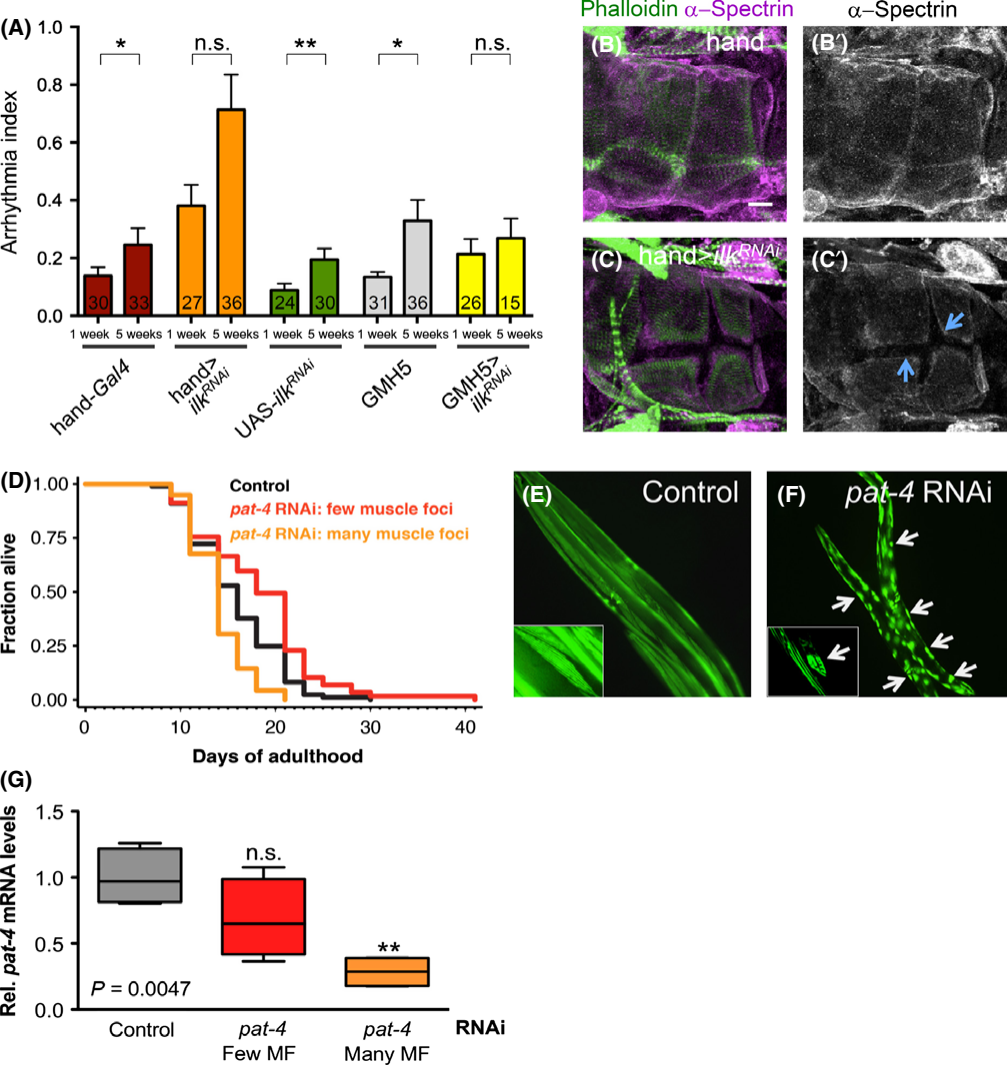
Effect of *ilk* RNAi knock-down (KD) in *Drosophila* hearts and of *ilk/pat-4* RNAi KD on *Caenorhabditis elegans* lifespan. (A) Bar graph representations of arrhythmia index (AI) of 1-week- and 5-week-old *ilk* RNAi hearts. Wild-type controls (hand-*Gal4*, UAS-*ilkRNAi* and GMH5) show the expected age-dependent increase in AI. Heart-specific *ilk* KD hearts (with hand-*Gal4*, orange bars) have an elevated AI at 1 week, which seemed to worsen with age (not statistically significant). In contrast, cardiomyocyte-specific *ilk* KD hearts (with GMH5, yellow bars) have a slightly elevated AI at 1 week, which did not exhibit a further increase at 5 weeks. Each sample number is indicated at the bottom of each bar. *P < 0.05, **P < 0.01, ***P < 0.001, Mann–Whitney test. Error bars are SEM. (B-C’) Heart structure in conical chamber region visualized by phalloidin staining (green in B and C) and anti-α-spectrin antibody (magenta in B and C, grayscale in B’ and C’). Heart-specific *ilk* RNAi caused gaps between cardiomyocytes (C, C’, arrows). Bar, 20 μm. (D–F) *C. elegans* expressing a GFP-tagged body-wall muscle myosin reporter (MYO-3::GFP) were fed control bacteria (vector only) or bacteria expressing *ilk/pat-4* dsRNA from hatching. (D) On day 1 of adulthood, animals were grouped according to either a low or a high number of detached cytoskeleton in muscle cells (referred to as few or many ‘muscle foci’, respectively, see Experimental procedures), and their lifespan was assayed. While control animals had a mean lifespan of 16 days (*N* = 104), *ilk/pat-4* (RNAi) animals with a low number of detached muscle cells had a mean lifespan of 18 days (*N* = 114), thus displaying a ~ 15% increase in mean lifespan (*P* < 0.001, log-rank test). In contrast, *ilk/pat-4 (RNAi)* animals in which almost all muscle cells were detached displayed a ~ 11% lifespan reduction (mean lifespan 14 days, *N* = 114, *P* < 0.005, log-rank test). This experiment was repeated twice with similar results. (E, F) Animals subjected to (E) control RNAi with few ‘muscle’ foci’ or to (F) *ilk/pat-4* RNAi with many visible ‘muscle foci’ were imaged on day 2 of adulthood at 50× magnification. Arrows point to examples of muscle cells with detached MYO-3::GFP structures, and inserts show higher magnification of single muscle cells. (G) The *ilk/pat-4* transcript levels of animals with a low or a high number of ‘muscle foci’ (MF) were determined using qRT-PCR. Only the animals with a high number of ‘muscle foci’ show a significant reduction in *ilk/pat-4* transcript levels (n.s. *P* > 0.05, **P < 0.005, one-way ANOVA).

Given the mixed results with *ilk* manipulations elsewhere (Chen *et al*., 2006; White *et al*., 2006), it is possible that *ilk* has multiple functions. Thus, we hypothesize *ilk* reduction could have detrimental or beneficial effects depending on the conditions. To ask whether *ilk* is also critical for maintaining heart function in *Drosophila*, we examined the effect of cardiac RNAi-mediated knockdown of *ilk* (using two different drivers, the strong heart-specific *hand-*Gal4 (that includes some pericardial cells) and the weaker cardiomyocyte-specific GMH5 (Wessells *et al*., 2004; Han & Olson, 2005). Strong cardiac reduction in *ilk* leads to high arrhythmias in young flies, which was further elevated at old age (orange bars in Fig. 5A). In contrast, weaker *ilk* KD in cardiomyocytes (see also qPCR in Fig. S3, Supporting information) prevented an increase in the level of arrhythmias with age (yellow bars in Fig. 5A), unlike in wild-type controls. This suggests that a moderate reduction in *ilk*, as in *ilk* heterozygotes (Fig. 3), averts an age-dependent increase in AI, but a more substantial reduction disrupts the regular beating patterns, including at young ages.

To investigate this notion further, we examined the structural integrity of hearts with strong cardiac KD of *ilk*. With hand-*Gal4*-mediated KD, we found severe morphological defects in *ilk* RNAi hearts, including gaps between cardiomyocytes and abnormal patterns of β1-integrin staining, which were not seen in wild-type controls or *ilk* heterozygous mutants (Fig. 5B–C, Fig. S4A–E, Supporting information). This suggests that ILK may be required for adhesion between cardiomyocytes, consistent with ILK localization at cell–cell contact sites in wild-type hearts (Fig. 2A–C). These results are in contrast to our finding with *ilk* heterozygous mutants, which showed improved cardiac performance at older age (Fig. 3). Thus, cardiac *ilk* KD with hand-*Gal4* is likely to cause more severe diminution of *ilk* function than the expected 50% reduction in function of *ilk* heterozygous mutants or with GMH5-mediated KD (see Fig. S3). Interestingly, heart-specific KD of *pinch*, encoding an ILK binding partner, results in similar intercellular adhesion defects (Fig. S4F-F”). In addition, heart-specific KD of *mys* or *talin*, the latter encoding a critical integrin–actin linker essential for integrin activation, also compromised cardiomyocyte adhesion to their neighbors (Fig. S4G–J). This finding supports the hypothesis that the integrin complex is essential for adhesion between adult cardiomyocytes.

### Dual effects of *ilk/pat-4* RNAi knockdown on *C. elegans* longevity

To investigate whether the dual – beneficial versus detrimental – role of scaled reduction in *ilk* signaling in aging is conserved over a substantial evolutionary distance, we turned to another model aging organism, *C. elegans*. Similar to *ilk* or *mys* null mutations in *Drosophila*, *C. elegans ilk/pat-4* null mutants are also embryonic lethal (MacKinnon *et al*., 2002). However, post-embryonic RNAi KD results in viable animals with extended lifespan, suggesting *ilk/pat-4* as an important and conserved longevity gene (Hansen *et al*., 2005; Curran & Ruvkun, 2007; Kumsta *et al*., 2014). However, long-lived animals exposed to *pat-4*/ILK RNAi since hatching are paralyzed, because of detached cytoskeleton of muscle cells (‘muscle foci’), which was visualized by GFP-tagged myosin heavy chain A/MYO-3 (Fig. 5E,F; Campagnola *et al*., 2002; Kumsta *et al*., 2014). This detachment phenotype is reminiscent of what is observed in *Drosophila ilk* homozygous embryos (Zervas *et al*., 2001). To test whether the lifespan extension was linked to the paralysis phenotype, we grouped populations of *C. elegans* with either mild or severe muscle cytoskeleton detachment in early adulthood (Fig. 5E,F) and then carried out lifespan analysis. Interestingly, we found that the population with many MYO-3-positive muscle foci was shorter-lived, whereas the group that had either no or few muscle foci was longer lived (Fig. 5D). To address whether this difference in the lifespan of animals correlated with the severity of the reduction in *ilk/pat-4,* we conducted qPCR to measure *ilk/pat-4* transcript levels in these animals. Indeed, animals with few muscle foci did not exhibit as dramatic a reduction in *ilk/pat-4* RNA levels as compared with animals with many muscle foci (Fig. 5G). Taken together, these observations suggest that a substantial reduction in *ilk/pat-4* is detrimental for whole organismal physiology and lifespan, whereas a milder reduction improves longevity of *C. elegans*, thus underlining a dual role in aging and muscle integrity that is evidently conserved in evolution.

### Dual effects of β1-integrin/ILK pathway on heart function

**Figure 6:**
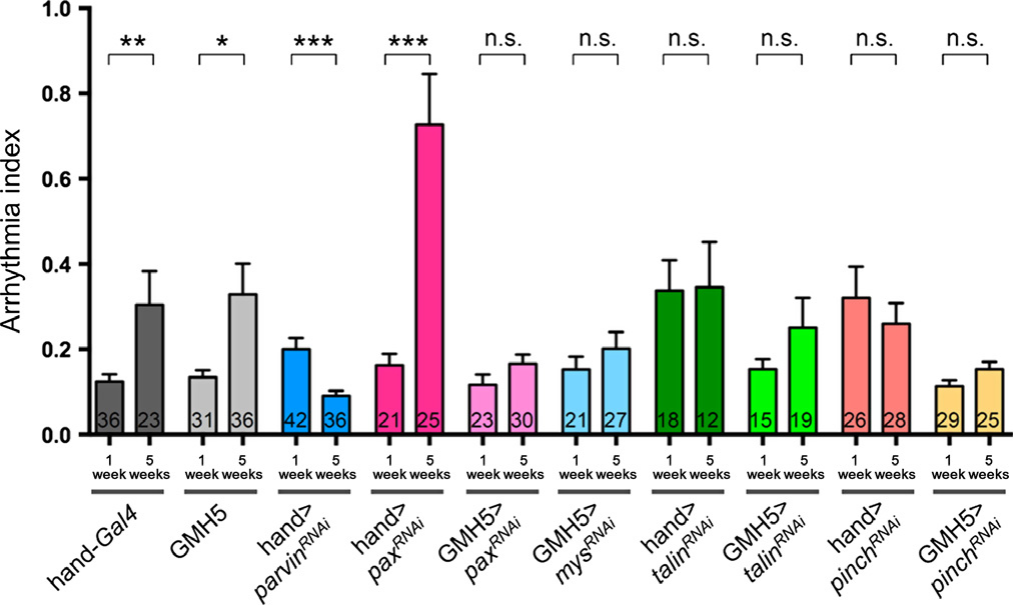
RNAi knock-down (KD) analysis of integrin-linked kinase/β1-integrin pathway components. Bar graph representations of the arrhythmia index (AI) of 1- and 5-week-old hearts with hand-*Gal4* and GMH5 mediated KD of *parvin, pax, mys, talin, and pinch*. Wild-type controls (hand-Gal4 and GMH5 drivers alone) show the expected age-dependent increase in AI. In contrast none of the experimental KDs exhibited an age-dependent increase in AI, except for hand > *pax*-RNAi, which was much elevated at 5 weeks. Moreover, strong hand-driven KD of *parvin, talin,* and *pinch* was causing a high AI already at 1 week. Each sample number is indicated at the bottom of each bar. Error bars indicate SEM. *P < 0.05, **P < 0.01, ***P < 0.001, Mann–Whitney test.

Given the beneficial or detrimental effects depending on the extent of *ilk* reduction, we wondered whether strong versus weak inhibition of ILK binding partners also could have opposing phenotypic effects on cardiac function in *Drosophila*. One of ILK’s binding partners is Parvin, an adaptor protein containing two calponin homology domains (Tu *et al*., 2001). In *C. elegans*, RNAi KD of *parvin* (*pat-6*) significantly extends lifespan, similar to *ilk* KD (Hansen *et al*., 2005). Thus, an appropriate reduction in *parvin* may also have beneficial effects on cardiac aging. When *parvin* was knocked down in the fly’s heart using hand*-Gal4*, the flies survived to adulthood and displayed modestly elevated arrhythmias at 1 week of age suggesting a slightly detrimental effect at that age (Fig. 6). However, at 5 weeks of age, the incidence of arrhythmias with *parvin* KD hearts was low, similar to the 1-week time point of wild-type control (hand-*Gal4* alone) and comparable to long-lived *ilk* and *mys* heterozygotes (Fig. 3 and Fig. S1) This suggests a beneficial effect of *parvin* KD on old hearts by preventing an age-dependent increase in AI. Examining *pax*, *talin,* and *pinch*, coding for other ILK-interacting proteins, we found that hand*-Gal4-*mediated KD exhibited high AI levels for *talin* and *pinch* already at young ages (Fig. 6), comparable to strong *ilk* KD (Fig. 5A), and consistent with the observed structural defects (Fig. S4). In contrast, moderate cardiomyocyte KD (using GMH5) of *pax, talin, pinch*, as well as *mys*, exhibited a low AI, typical of 1-week control heart and importantly failed to show any significant age-dependent elevation of AI, unlike their wild-type controls (Fig. 6), thus strongly suggesting a similar beneficial effect of fine-tuned reduction in gene function as with *ilk* or *mys*. Interestingly, weak *talin* KD did not show increased arrhythmias at young ages, but already resulted in some disorganization in myofibrillar structure (Fig. S4I,J).

To further substantiate that moderate reduction in ILK pathway components in the myocardium contributes to better cardiac performance at old ages, we examined heterozygous mutants of *parvin, pinch* (*stck*), and *talin* (*rhea*; see Experimental procedures). All three heterozygotes failed to show an increase in AI or decrease in diastolic diameter at old ages (Fig. S5, Supporting information), thus corroborating the conclusion that they indeed participate in the beneficial modulation of cardiac aging, similar to moderate *ilk* and *mys* attenuation (Figs 3 and 5A). Together, these results further support the notion that moderately reduced levels of β1-integrin/ILK signaling components within the heart prevent the increase in cardiac arrhythmias with age, whereas strong reduction causes severe functional and structural defects often already at young ages.

## Discussion

Our data demonstrate that the β1-integrin/ILK pathway is a critical genetic modulator of cardiac and organismal aging. We have shown that reduction in β1-integrin/ILK levels is beneficial for *Drosophila* longevity and cardiac performance at older ages. In addition, severe reduction in ILK pathway components in the heart causes loss of cardiomyocyte cell adhesion, along with structural and functional deficits. We also find that β1-integrin protein levels increase with age in wild-type flies and that overexpression of *mys* seems to be detrimental to the heart and perhaps causes a premature aging-like phenotype in the heart at young ages. Whether this is a *bona fide* progeric phenotype awaits further study. However, considering that *ilk* KD also extends *C. elegans* lifespan (Hansen *et al*., 2005; Curran & Ruvkun, 2007; Kumsta *et al*., 2014), mutation in *mys* increases *Drosophila* lifespan (Goddeeris *et al*., 2003) and that the inhibition of *ilk* expression prevents senescence in old rat fibroblasts (Chen *et al*., 2006), we suggest that the role of integrin/ILK pathway components in (cardiac/organismal) aging is likely conserved across species.

Similar to our findings, it has been shown that β1-integrin is significantly higher in old compared with young monkey vascular smooth muscle cells (VSMCs; Qiu *et al*., 2010). Therefore, the accumulation of β1-integrin might be a common phenomenon underlying both myocardial and vascular aging. Interestingly, applied mechanical force appears to regulate the assembly of focal contacts and integrin turnover (Riveline *et al*., 2001; Pines *et al*., 2012), consistent with the idea that with age this regulation is no longer as finely tuned leading to excess integrin accumulation and consequent heart defects.

In contrast to the cardioprotective effects of moderate reductions in integrin/ILK complex levels, more drastic interference with complex function/levels can lead to severe abnormalities in cardiac structure and function, suggesting a critical requirement of this complex across species. In mice, *ilk* ablation causes dilated cardiomyopathy, heart failure, and sudden death (White *et al*., 2006). In flies, strong cardiac *ilk* complex KD causes severe arrhythmia and loss of cardiac integrity, including gaps between adjacent cardiomyocytes. In contrast, the moderate reduction in *ilk* complex function attenuates the age-dependent changes in cardiac performance, suggesting that alterations in this pathway need to be finely tuned to have beneficial effects, and this seems to be the case across species.

Interestingly, similar observations have been made for ion channel function: Expression of KCNQ, encoding a K^+^ channel responsible for repolarizing the cardiac action potential, is reduced at old age (Nishimura *et al*., 2011). Heart-specific overexpression of KCNQ in old wild-type flies reverses the age-dependent increase in arrhythmia, whereas overexpression of KCNQ in young flies increases the incidence of arrhythmias (Ocorr *et al*., 2007; Nishimura *et al*., 2011). Thus, precise control of signaling via KCNQ channels, as well as via the integrin/ILK pathway investigated here, seems to be critical for tipping the balance between beneficial and detrimental effects.

Integrins can mediate both ‘outside-in’ and ‘inside-out’ signaling and interactions (Legate *et al*., 2006). For ‘outside-in’ signaling, integrin activation recruits a large variety of proteins that interface with the actin cytoskeleton and affect many diverse signaling pathways, including ILK-mediated phosphorylation of target genes, such as AKT. However, the requirement for the kinase activity of ILK has been questioned (Wickström *et al*., 2010), and the longevity effect of *ilk/pat-4* RNAi appears to be largely *foxo/daf-16* independent in *C. elegans* (Hansen *et al*., 2005; Curran & Ruvkun, 2007). Interestingly, the longevity effect of *ilk/pat-4* RNAi in *C. elegans* instead seems to be dependent on heat shock factor HSF-1 (Kumsta *et al*., 2014). Further investigation is necessary to address whether this is also the case in *Drosophila*.

With ‘inside-out’ signaling, integrin activation might modulate the assembly of ECM ligands on the cell surface. Excessive deposition of ECM is associated with ventricular fibrosis that leads to stiffening of the ventricular wall causing ventricular dysfunction and heart failure (Pellman *et al*., 2010). In *Drosophila*, β1-integrin protein levels are increased with age (Fig. 4A,B) and *mys* heterozygous mutants lack the age-dependent increase in myocardial stiffness (Fig. 3F). Therefore, it is possible that increased levels of β1-integrin during aging causes excessive ECM deposition leading to stiffening of the heart, which results in diastolic dysfunction and an elevated incidence of arrhythmias with age.

Although in the aggregate our results show that a moderate decrease in several components of β1-integrin/ILK signaling can reverse several characteristics of cardiac aging, it is interesting that there are notable exceptions. For example, *mys* heterozygotes in the same genetic background as *ilk* do not reverse the age-dependent decrease in diastolic diameter (Fig. 3C) and thus still show an age-dependent diastolic dysfunction. This may reflect a difference in the functional spectrum of the different signaling components that is not only and completely dedicated to this signaling pathway. Alternatively, an occasional inconstancy may also be due to experimental variability inherent in these types of physiological experiments.

In conclusion, our data provide novel insights into cardiac-specific aging involving a recently identified pathway that modulates organismal aging: the integrin/ILK complex. We find that to maintain a youthful cardiac and organismal physiology, the pathway’s activity needs to be finely tuned and maintained at specific activity levels, and this set point appears to be altered or deregulated during aging. Thus, the integrin/ILK pathway emerges as a key modulator of cardiac homeostasis and aging.

## Experimental procedures

### Fly strains

*ilk*^*54*^ (Zervas *et al*., 2011; the allele *ilk*^*54*^ has a premature stop codon instead of a codon for W7), ILK-GFP;*ilk*^*54*^ (ILK-GFP is previously described in Zervas *et al*., 2001), and UAS-*mys*^*wt*^ were generous gifts from Y. Inoue. *ilk*^*1*^ (Zervas *et al*., 2001) and *mys*^*1*^ (Bunch *et al*., 1992) were obtained from the *Drosophila* stock center. *w*^*CS*^ (Cook-Wiens & Grotewiel, 2002) and *mys*^*XG43*^ (Goddeeris *et al*., 2003) were kindly provided by M. Grotewiel. The *mys*^*XG43*^ allele is a 113-bp deletion in exon 5 causing a frame shift and premature truncation of *mys*, and introgressed into the *w*^*CS*^ genetic background for six generation (Goddeeris *et al*., 2003). *ilk*^*54*^ was introgressed into the *w*^*CS*^ genetic background for six generation in this study. The following RNAi lines were obtained from Vienna Drosophila RNAi Center (VDRC): UAS-*parvin*^*RNAi*^ (#11670), UAS-*pinch*^*RNAi*^ (#52538), UAS-*mys*^*RNAi*^ (#29619), UAS-*talin*^*RNAi*^ (#40399) and UAS*-paxillin*^*RNAi*^ (#107789). UAS-*ilk*^*RNAi*^ (originally from VDRC, #16062) was kindly given by F. Schnorrer. The effectiveness of *ilk, mys, parvin,* and *talin* RNAi lines had been tested in Schnorrer *et al*. (2010) and found lethal when crossed to the mesodermal driver mef2-Gal4. We have also tested ourselves the *pinch* and *parvin* RNAi lines when crossed to mef2-Gal4 and found them lethal as well. In addition, Perkins *et al*. (2010) tested *mys* and *talin* RNAi KD effectiveness. Cardiac-specific driver hand-*Gal4* and cardiomyocyte-specific driver GMH5 have been previously described (Wessells *et al*., 2004; Han & Olson, 2005). *parvin*^*694*^ (Vakaloglou *et al*., 2012), *stck*^*T2*^ (Zervas *et al*., 2011), and *rhea*^*79A*^ (Brown *et al*., 2002) were kindly provided by C. Zervas.

### Immunostaining

Adult female flies were dissected and immunostained as previously described (Taghli-Lamallem *et al*. 2008). Images were acquired using ApoTome (Carl Zeiss Microscopy, Thornwood, NY, USA) or FV1000 (Olympus, Center Valley, PA, USA). The following primary antibodies were used: rabbit anti-GFP (1:250, Invitrogen, Grand Island, NY, USA), mouse anti-αPS (1:20, CF.6G11; DSHB Iowa), mouse anti-α-actinin (1:20, kindly provided by J. Saide), and mouse anti-α-spectrin (1:50, 3A9; Developmental Studies Hybridoma Bank, University of Iowa, Iowa City, IA, USA).

### Lifespan assays

Virgin female and male progeny were collected for 3 days. Then, they were briefly anaesthetized and separated in groups of 25 flies in each vial. The flies were kept at 25 °C, and the dead flies were counted every 3 days after transfer. Each experiment was performed twice: first on the smaller scale (100–150 flies) and then on larger scale (200–250 flies). Data were analyzed using Prism 5.0 (GraphPad Software, La Jolla, CA, USA).

### *C. elegans* RNAi treatment and lifespan assay

The *C. elegans* strain RW1596: *myo-3 (st386)V; stEx30 [myo-3p::gfp::myo-3 + rol-6]* (Campagnola *et al*., 2002) used in this study was maintained and cultured under standard conditions at 20 °C using *Escherichia coli* OP50 as a food source, except when subjected to RNAi treatment. The *pat-4* RNAi clone was obtained from the Ahringer RNAi library. RNAi treatment was carried out as previously described (Hansen *et al*., 2005). Muscle detachment was scored on day 1 and day 2 of adulthood under a fluorescent stereoscope, and animals were grouped into two groups based on the efficiency of the RNAi treatment – one group had 0–3 detached muscle cells per animal, whereas the other group had a high degree of detachment (> 80% detached muscle cells per animal). Lifespan assays were carried out as previously described (Hansen *et al*., 2005). For statistical analysis, Stata software was used (StataCorp, College Station, TX, USA). *P* values were calculated with the log-rank (Mantel–Cox) method.

### Heartbeat analysis

Flies were anesthetized with fly nap (Carolina Biological Supply Co., Burlington, NC, USA) and dissected as previously described (Ocorr *et al*., 2007). Movies were taken at rates between 140 and 160 frames per second for 30 s using a Hamamatsu CCD digital camera (McBain Instruments, Chatsworth, CA, USA) on a Leica DM LFSA microscope with a 10× water immersion lens and HCImage imaging software. The images were analyzed, and M-modes were generated using Semi-automatic Optical Heart Beat Analysis software (Fink *et al*., 2009).

### *Drosophila* heart indentation

Adult female flies were anesthetized and immobilized on 25-mm glass coverslips with a thin layer of vacuum grease ventral-side-up. The heart tube was exposed via microsurgery as previously described (Ocorr *et al*., 2007) with additional micropipette aspiration to remove all ventral tissue proximal to the conical chamber. Each coverslip is mounted on a Fluid Cell Lite coverslip holder (Asylum Research, Goleta, CA, USA) with 1 mL of hemolymph. Hearts are checked for regular contractions to ensure they are in good health and then resubmerged in 10 mm ethylene glycol tetraacetic acid (EGTA)-treated hemolymph to arrest contraction. Prior to indentation, probes were calibrated via thermal noise method in MFP-3D Bio software (Asylum Research). Nanoindentation was performed on an MFP-3D Bio Atomic Force Microscope (Asylum Research) mounted on a Ti-U fluorescent inverted microscope (Nikon Instruments, Melville, NY, USA) with 120 pN nm^−1^ silicon nitride cantilevers with premounted, 2-μm radius borosilicate glass spheres (Novascan Technologies, Ames, IA, USA). All indentation curves were analyzed to calculate myocardial stiffness using previously published, automated software custom-written in MATLAB (MathWorks, Natick, MA, USA; Kaushik *et al*., 2011, 2012).

### Statistical methods

Statistical Analyses were performed using Prism 6.0 (Graph Pad Software, Inc.). All data were checked prior to analysis using the D’Agostino & Pearson omnibus normality test to determine whether the data violated the assumption of a Gaussian distribution. We employed a one-way analysis of variance (ANOVA) when comparing more than two groups (e.g., Fig. 4D,E) followed by Tukey’s multiple comparisons *post hoc* test. If the data were not normally distributed (e.g., Fig. 4C), a Kruskal–Wallis test was performed followed by Dunn’s multiple comparisons *post hoc* test. A two-way ANOVA was employed when comparing two or more groups at more than one age (e.g., Fig. 3B–E). Data that did not exhibit a Gaussian distribution (the arrhythmia indices) were first normalized before applying a two-way ANOVA (e.g., Fig. 3B). *Post hoc* comparisons of two-way ANOVA analyses were made using a Dunnett’s multiple comparisons test. Comparisons of arrhythmia indices within a single genotype between two ages were made using the Mann–Whitney test for nonparametric data (e.g., Fig. 5A). Lifespan analyses were performed using a log-rank analysis (Mantel–Cox test). Specific tests used are indicated in the figure legends. In all cases, *P* values less than 0.05 were taken as significant.

## Supporting Information

**Fig. S1** *ilk* heterozygotes and *mys* heterozygotes retard cardiac decline with age. **Fig. S2** mRNA levels of *ilk* and *mys* do not change with age. **Fig. S3** *ilk* expression is reduced by cardiac RNAi knockdown. **Fig. S4** *pinch*, *mys*, and *talin* RNAi but not *ilk* heterozygous mutants induce gaps between cardiomyocytes. **Fig. S5** Heterozygous mutants for *parvin, pinch and talin* lack age-dependent heart function changes.
