# Supplementary material for: A dual role for integrin-linked kinase and β1-integrin in modulating cardiac aging
Source: Aging Cell. 2014 Jan 9;13(3):431–40. doi: 10.1111/acel.12193 (PMC4032615; doi:10.1111/acel.12193)
Supplement: Supplementary file 1 — Fig. S1 ilk heterozygotes and mys heterozygotes retard cardiac decline with age. Fig. S2 mRNA levels of ilk and mys do not change with age. Fig. S3 ilk expression is reduced by cardiac RNAi knockdown. Fig. S4 pinch, mys, and talin RNAi but not ilk heterozygous mutants induce gaps between cardiomyocytes. Fig. S5 Heterozygous mutants for parvin, pinch and talin lack age-dependent heart function changes. [file acel0013-0431-sd1.pdf]

Supporting Information

Supplementary Figure 1: *ilk* heterozygotes and *mys* heterozygotes retard cardiac decline with age.

Supplementary Figure 2: mRNA levels of *ilk* and *mys* do not change with age.

Supplementary Figure 3: *ilk* expression is reduced by cardiac RNAi knockdown.

Supplementary Figure 4: *pinch*, *mys*, and *talin* RNAi but not *ilk* heterozygous mutants induce gaps between cardiomyocytes.

Supplemental Figure 5. Heterozygous mutants for *parvin, pinch and talin* lack age-dependent heart function changes.

qRT-PCR

Drosophila: RNA was extracted from 20-30 hearts using PicoPure™ RNA Isolation Kit (ARCTURUS). The samples were treated with DNaseI (Qiagen) to remove DNA. First strand cDNA was synthesized using SuperScript™ III First-Strand Synthesis System for RT-PCR (Invitrogen). Polymerase chain reactions were performed using the LightCycler FastStart DNA Master^{PLUS} SYBR Green I (Roche Applied Science). Three technical replicates of triplicate biological samples were analyzed for each genotype. The following primers were used: *ilk* forward: CTGACGGCGAGAATCAACATG, *ilk* reverse: ATGTGCCTGGCGGAATCTTG, *mys* forward: GAATGCGGTG TCTGCAAGTG, *mys* reverse: TTCCTGGCGCAATCATCACCGTTC, *tubulin84B* forward: GCTGTTCCACCCCGAGCAGCTGATC, *tubulin84B* reverse: GGCGAACTCCAGCTTGGACTTCTTGC, *rpl32* forward: GTGCGCTTGTTCGATCCGTAACCG, *rpl32* reverse: CTAAGCTGTCGCACAAATG.

C. elegans: Total RNA was isolated from a mixed population of ~2000 nematodes (RW1596: *myo-3(st386)V; stEx30[myo-3p::gfp::myo-3 + rol-6]*, grown on NGM plates seeded with control bacteria (vector only) or bacteria expressing *pat-4* dsRNA. To induce a strong muscle detachment phenotype, IPTG was added to the plates to ensure robust *pat-4* dsRNA expression, whereas mild muscle detachment was achieved by the

leaky expression of the dsRNA by the T7 promoter without induction by IPTG. The severity of the muscle detachment was verified prior to harvesting under a fluorescent stereoscope. For RNA extraction the animals were flash frozen in liquid nitrogen. RNA was extracted using Trizol (Life Technologies) and purified using the Qiagen RNeasy kit (Valencia, CA), including an additional DNA digest step using the Qiagen Dnase I kit. The M-MuLV reverse transcriptase and random 9-mer primers (New England) were used for reverse transcription of 1 μg of RNA per sample. Quantitative PCR was run using SYBR Green Master Mix in a Roche LC480 LightCycler (Indianapolis, IN). A standard curve was run for each primer on serial dilutions of a mixture of different cDNAs, and the observed CT values were converted to relative values according to the primers' standard curves. The mRNA levels of target genes were normalized to those of the housekeeping gene nuclear hormone receptor *nhr-23*. Three biological replicates (with three technical replicates) were carried out. The following primers were used: *ilk/pat-4* forward: CCTATTCGCCTGCCTGGAT, *ilk/pat-4* reverse: CATATTACGGGCGATTCCAG, *nhr-23* forward: CAGAAACACTGAAGAACGCG, *nhr-23* reverse: CGATCTGCAGTGAATAGCTC

Western blot analysis

Four to five whole bodies from age-matched control, *ilk*^{54}/+, and *mys*^{XG43}/+ females were homogenized in lysis buffer: 10mM Tris (pH7.8), 1% NP40, 0.15M NaCl, 4mM EDTA, 10% glycerol, 1x proteinase inhibitor (Roche Applied Science). The lysates were clarified by centrifugation, quantified with Bradford Reagent (Bio-rad), diluted with Laemmli Sample Buffer (Bio-Rad) containing 5% β-mercaptoethanol, and boiled. After separation by SDS-PAGE, they were transferred on a cellulose nitrate membrane (Invitrogen). The blots were blocked with 5% skim milk/TBS (150mM NaCl, 20mM Tris) and proved with mouse anti-Tubulin (1:15000, Sigma) and rabbit anti-αPS (1:1000, kindly provided by Richard Hynes, MIT, USA) in 5% skim milk/TBST (0.05% Tween20/TBS). Fluorescently-labeled secondary antibodies (Li-Cor Bioscience, Lincoln, NE, USA) in Odyssey blocking buffer (Li-Cor Bioscience) were added and the fluorescence was detected and quantified by using Odyssey Infrared Imaging System (Li-CorBioscience).

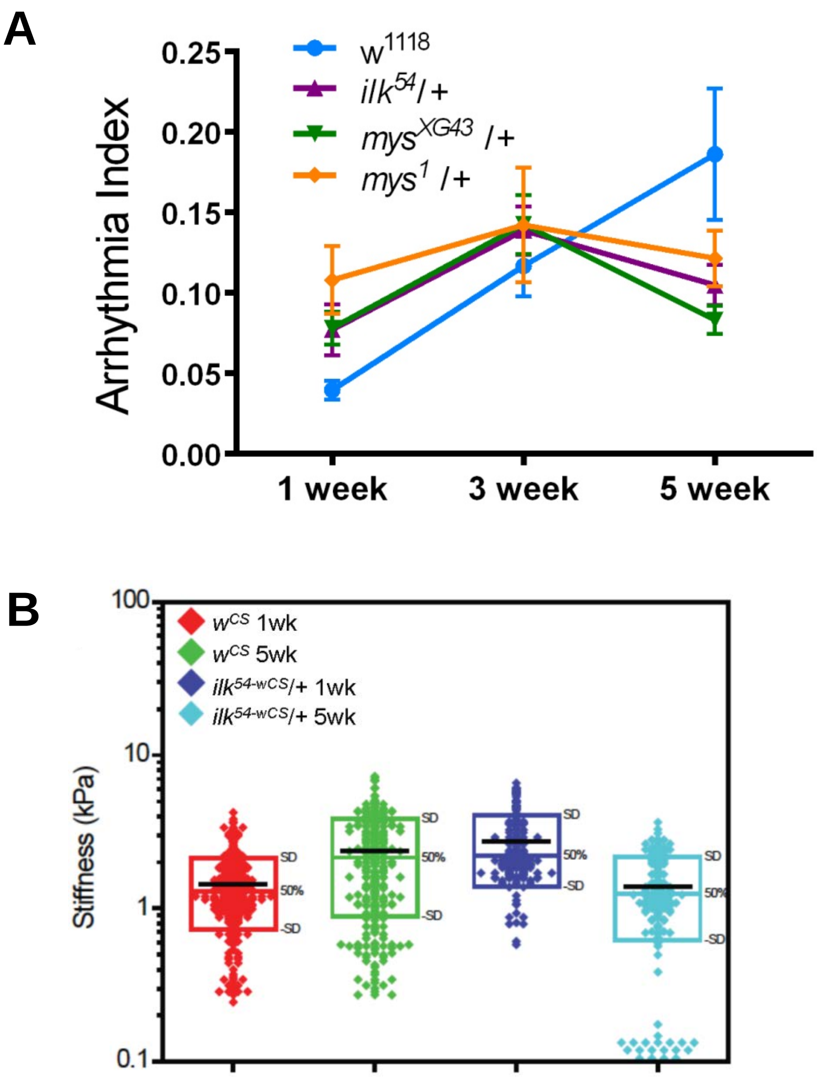

Supplementary Figure 1: *ilk* heterozygotes and *mys* heterozygotes retard cardiac decline with age. Heart physiology was examined in w^{1118}(●),ilk^{54}/+(▲), mys^{XG43}/+(▼), and mys^{1}/+(◆) flies at 1, 3, and 5 week that have been crossed out to w^{1118}. 20-50 flies were used for each data point. **(A)** Plots of Arrhythmia Index. w^{1118} flies showed the increase of arrhythmia with age, but the increase was significantly reduced or abolished in ilk^{54}/+, mys^{1}/+ and mys^{XG43}/+ at 5 weeks. Two-way ANOVA, *p<0.05, ***p<0.001. **(B)** Scatter plot of the data shown in Figure 3F to indicate intra-genotype and age-dependent variation in heart tube stiffness.

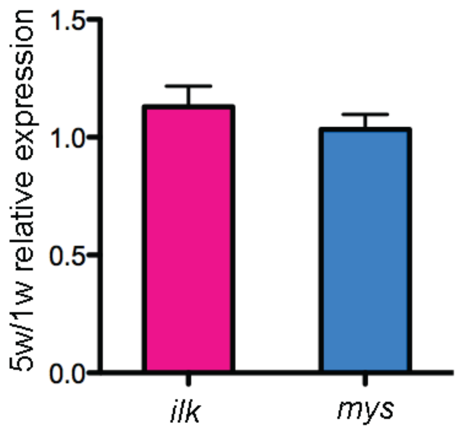

Supplementary Figure 2: mRNA levels of *ilk* and *mys* do not change with age. The mRNA levels of *ilk* and *mys* isolated from dissected hearts were examined by qRT-PCR. Three biological samples were analyzed and normalized to *tubulin84B* expression. Relative expression of *ilk* or *mys* at 5-week did not change compared to those at 1-week. Error bars: SEM.

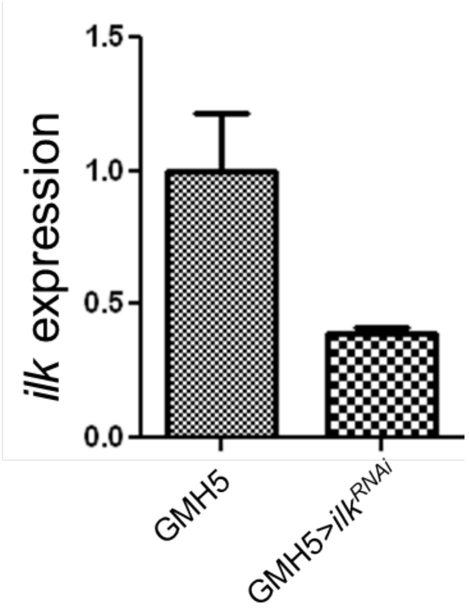

Supplementary Figure 3: *ilk* expression is reduced by cardiac RNAi knockdown. *ilk* mRNA levels were examined by qRT-PCR of 3 biological samples normalized to *Rp49* expression. GMH5-induced *ilk* RNAi decreased *ilk* mRNA levels in the heart by about half, compared to GMH5 (Gal 4 cardiomyocyte-specific driver) alone, which served as the wildtype control.

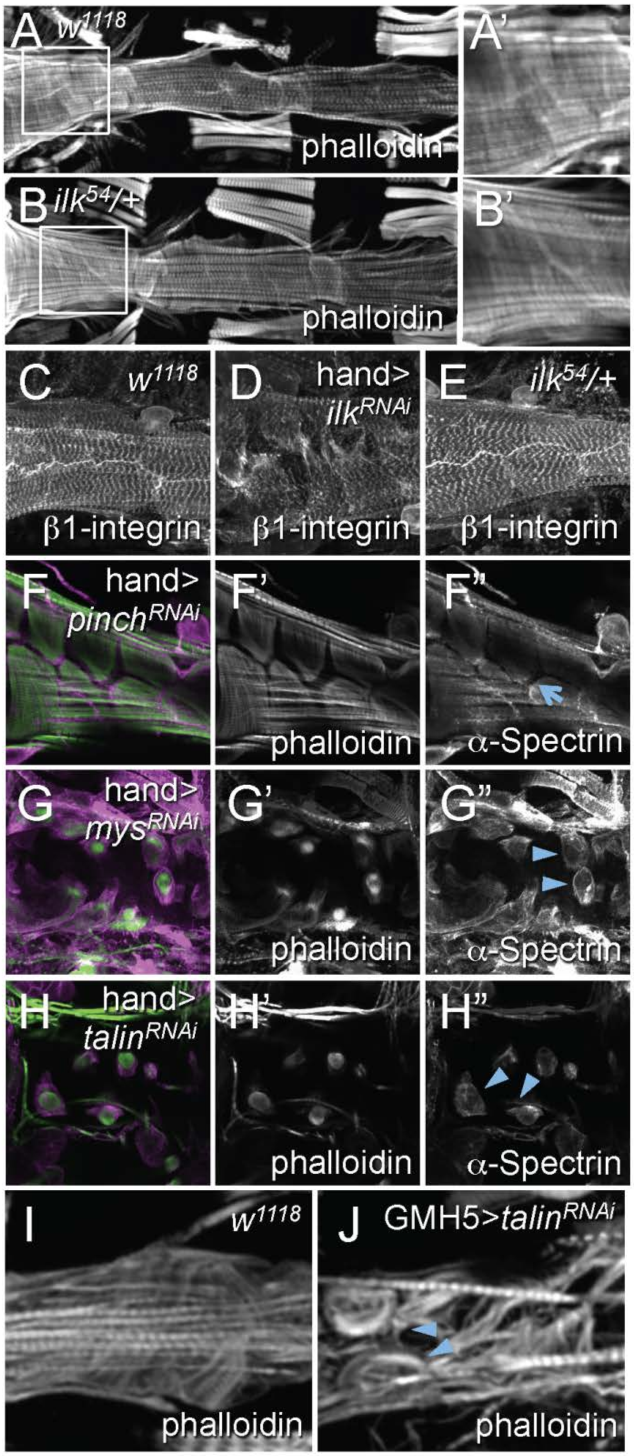

Supplementary Figure 4: *pinch*, *mys*, and *talin* RNAi but not *ilk* heterozygous mutants induce gaps between cardiomyocytes. Heart structure was visualized by phalloidin staining (gray-scale in A-B', F', G', H', I, and J, green in F, G, and H) and anti-α-Spectrin antibody (magenta in F, G, and H, gray-scale in F", G", and H") or anti-β1-Integrin antibody (C-E). **(A-B')** The regions from abdominal segment 2 to 4 are shown in A and B. A' and B' are higher magnification images of the conical chamber regions shown in square in A and B, respectively. **(A, A")** Heart structure of 1-week old w^{1118} flies. **(B, B")** Heart structure of 1-week old ilk^{54}/+ did not show abnormality, such as gaps between cardiomyocytes. **(C-H")** The regions from abdominal segment 2 from 1-week old flies are shown. β1-Integrin patterns in 1-week old ilk^{54}/+ (E) was not obviously different from those in 1-week old w^{1118} flies (C), whereas heart-specific *ilk* RNAi impaired the patterns (D). **(F-F")** Heart-specific *pinch* RNAi caused gaps between cardiomyocytes (arrows). **(G-G")** Heart-specific *mys* RNAi resulted in ball-like cardiomyocytes losing their connections with the neighbors (arrowheads). **(H-H")** Heart-specific *talin* RNAi showed the similar defects to *mys* RNAi (arrowheads). **(I-J)** The regions from abdominal segment 3 are shown. 1-week old flies with *talin* RNAi expressed in the heart caused gaps between cardiomyocytes (J, arrowheads).

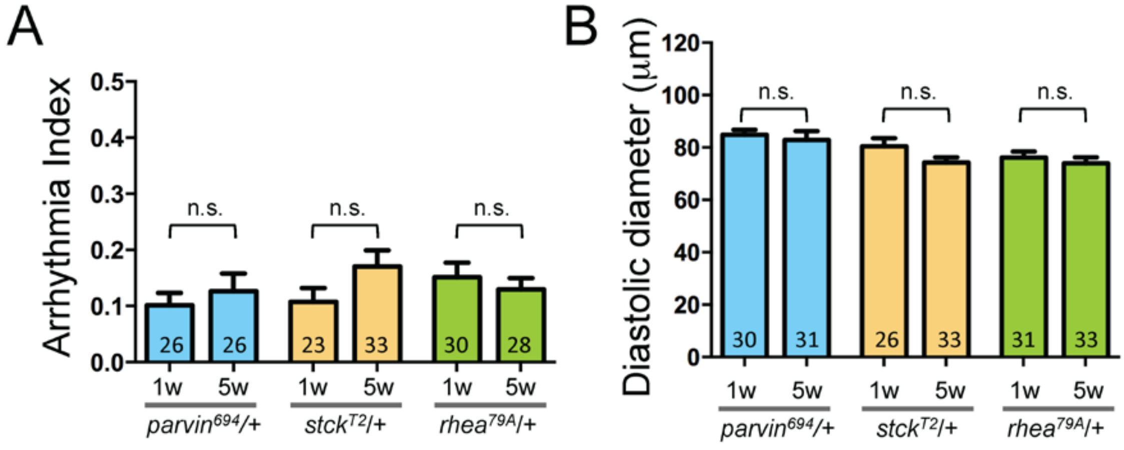

Supplementary Figure 5. Heterozygous mutants for *parvin, pinch and talin* lack age-dependent heart function changes. Bar graph representations of Arrhythmia Index **(A)** and Diastolic Diameter **(B)** at 1 and 5 week of *parvin*^{694}/+, *stck*^{T2}/+ (*pinch*) and *rhea*^{79A}/+ (*talin*). The heterozygotes do not exhibit elevated arrhythmias or a decrease in diastolic diameter with age. ($P>0.05$, Mann Whitney test). Each sample number is indicated at the bottom of each bar. Error bars indicate SEM.
